# The Epidemiology of Imported Malaria in Taiwan between 2002–2013: The Importance of Sensitive Surveillance and Implications for Pre-Travel Medical Advice

**DOI:** 10.3390/ijerph110605651

**Published:** 2014-05-27

**Authors:** Shou-Chien Chen, Hsiao-Ling Chang, Kow-Tong Chen

**Affiliations:** 1 Department of Family Medicine, Da-Chien General Hospital, Miaoli 350, Taiwan; E-Mail: scchen818@yahoo.com.tw; 2 General Education Center, Ta Tung University, Taipei 111, Taiwan; 3 Division of Surveillance, Centers for Disease Control, Taipei 104, Taiwan; E-Mail: hlchang@cdc.gov.tw; 4 School of Public Health, National Defense Medical Center, National Defense University, Taipei 117, Taiwan; 5 Department of Occupational Medicine, Tainan Municipal Hospital, Tainan 701, Taiwan; 6 Department of Public Health, College of Medicine, National Cheng Kung University, Tainan 701, Taiwan

**Keywords:** malaria, travel medicine, immigrant, imported case

## Abstract

The purpose of this study was to assess the epidemiology of imported malaria in Taiwan between 2002 and 2013. We analyzed the national data recorded by the Taiwan Centers for Disease Control (Taiwan CDC). Malaria cases were diagnosed by blood films, polymerase chain reaction, or rapid diagnostic tests. The risk of re-establishment of malarial transmission in Taiwan was assessed. A total of 229 malaria cases were included in our analysis. All of the cases were imported. One hundred and ninety-two cases (84%) were diagnosed within 13 days of the start of symptoms/signs; 43% of these cases were acquired in Africa and 44% were acquired in Asia. *Plasmodium falciparum* was responsible for the majority (56%) of these cases. Travel to an endemic area was associated with the acquisition of malaria. The malaria importation rate was 2.36 per 1,000,000 travelers (range 1.20–5.74). The reproductive number under control (R_c_) was 0. No endemic transmission of malaria in Taiwan was identified. This study suggests that a vigilant surveillance system, vector-control efforts, case management, and an educational approach focused on travelers and immigrants who visit malaria endemic countries are needed to prevent outbreaks and sustain the elimination of malaria in Taiwan.

## 1. Introduction

Malaria is a major global infection present in 108 countries inhabited by approximately 3 billion people; and causes approximately 216 million infections and 655,000 deaths worldwide annually [1]. It also causes avoidable deaths every year from imported malaria in non-endemic countries, mainly in people who are otherwise healthy [2]. The risks of contracting malaria vary over time because of changes in the epidemiology of malaria, changes in travel habits and patterns of migration, and the development of drug resistance [3,4]. The risk of infection during travel can be reduced by the use of anti-malaria prevention measures (AMPM) (e.g., wearing long-sleeve clothing and pants at night that provide full coverage, using insect repellents, sleeping under insecticide-impregnated bed nets (IIBN), and taking an appropriate chemoprophylaxis) [5,6,7]. The extent to which these measures are adopted depends on how well a traveler recognizes and understands the risks [8]. 

The resurgence of malaria in many of the areas in which it was previously eliminated during the Global Malaria Eradication Programme serves as a reminder that vigilant systems need to be sustained for as long as the mosquito vectors, a suitable climate and other conditions exist to facilitate disease transmission [9]. The risk of resurgence is determined by the prevailing vectorial capacity (receptivity), the malaria importation rate (vulnerability), and the malariogenic potential [10,11,12]. Therefore, malaria elimination, once achieved, is more likely to be sustained in regions where receptivity is low or decreased by human development or which are geographically isolated with limited movement across borders and limited importation of parasites [13,14]. 

Taiwan is located at 23°4' N and 121°0' E and has a subtropical climate. Temperatures range from cool to hot, and the humidity is relatively high throughout the year. Malaria is documented to have been prevalent throughout Taiwan during the 19th and 20th centuries. The maximum estimated number of cases was 1.2 million in 1952 [15,16]. During the late 1960s, a combination of improved housing and socioeconomic conditions, environmental management, an intensive program of residual spraying with DDT in Taiwan carried out over a period of 5 years, and case management reduced malaria morbidity to a very low level [17,18]. In November 1965, the World Health Organization (WHO) certified Taiwan as an area where malaria had been eradicated [19]. Since then, malaria case surveillance has been maintained to detect locally acquired cases, which could indicate the reintroduction of transmission, and to monitor patterns of resistance to antimalarial drugs. Imported malaria cases have been diagnosed in Taiwan for the past four decades. The majority of the cases were imported from endemic countries [20], and a few cases were contracted at medical facilities [21]. Imported malaria has been an increasing problem in Taiwan and Western countries in the last two decades. Possible reasons for this increase in imported malaria include the increase in the number of travelers to tropical countries and the growing number of immigrants from malaria-endemic countries [22,23,24]. By the end of 2011, an estimated 460,000 permanent immigrants resided in Taiwan (not including foreign laborers): 67% were from China, 19% were from Vietnam, 6% were from Indonesia, 2% were from Thailand, and 6% were from other countries [25]. These four countries are considered malaria-endemic areas [26].

To identify trends and risk groups, we analyzed the surveillance data for all malaria cases in Taiwan from 2002 to 2013. We compared the data with information available on the number of travelers and the *An. minimus* mosquito distribution in Taiwan to determine whether these data could be useful for improving the existing surveillance system and pre-travel recommendations. 

## 2. Methods

### 2.1. Surveillance of Malaria in Taiwan

Since 1990, the National Notifiable Diseases Surveillance System (NNDSS) has reported malaria cases to the Center for Disease Control of Taiwan (Taiwan CDC) [27]. Malaria is a reportable disease in Taiwan. Physicians are required to report all cases of malaria by entering the data into local databases and electronically forwarding the data to the Taiwan CDC within 24 h of case ascertainment using Taiwan CDC-developed software [28]. According to surveys administered in Taiwan [29], more than 84% of physicians would report notifiable diseases to the CDC if they diagnosed the disease in a patient. After the reports were received by the CDC, an epidemiologic team (field epidemiologist, entomologist, public health nurse) was assigned to perform a patient follow-up, verify the diagnosis and complete patient information. Follow-up consisted of in-person interviews, telephone calls and correspondence with health care providers as well as an interview with the patient. Collected information included the patient’s age, gender, area of residence, geographic location of exposure, personal contact, and travel history [30]. The information was obtained with the patient’s permission by an epidemiologic team using a structured questionnaire. Institutional review board approval for this study was obtained from the National Cheng Kung University Hospital, and informed consent was obtained from all patients or their parents from 2002 to 2013. 

### 2.2. Travel Data

The number of travelers was obtained from the Tourism Bureau, Ministry of Transportation and Communication, Taiwan (TBMTC) [31]. The TBMTC data included the annual numbers of overnight leisure trips abroad by destination country and the number of overnight trips to malaria-endemic countries between 2002 and 2013. The number of travelers from Taiwan to the destination countries was determined based on embarkation/disembarkation cards and travel agency reports completed for immigration and tourism purposes. 

### 2.3. Mosquito Data

Mosquito survey data for Taiwan was obtained from the Taiwan CDC [32]. From April to September for each year from 2003 and 2006, two to three villages were surveyed each month. On each visit, a larval survey was conducted using 14-cm diameter dippers along the banks of streams and ditches around or in the surveyed village. Two teams collected adult mosquitoes along the bank and its surroundings for 1 h between 10:00 and 12:00. All of the collected mosquitoes were stored in a dry ice box and brought back to the laboratory for species identification. Blood-fed mosquitoes were kept at −20 °C for blood meal identification [15,32]. 

### 2.4. Definitions

A malaria case was defined as a person with a laboratory-confirmed *Plasmodium* infection between 2002 and 2013. The laboratory confirmation indicates that malaria parasites were identified either by microscopic examination of a blood film or by PCR that was subsequently confirmed by microscopy [33,34]. 

Elimination of malaria was defined as the interruption of local mosquito-borne malaria transmission in a defined geographical area (*i.e.*, zero incidence of locally contracted cases), even though imported cases continued to occur. Therefore, continued intervention is required [35]. 

Vulnerability, or the malaria importation rate, was defined as either proximity to a malarious area or a frequent influx of infected individuals, groups, or infective anophelines [11]. 

### 2.5. Statistical Analysis

The malaria importation rate was calculated by dividing the number of imported cases reported to the Taiwan CDC by the Tourism Bureau, Ministry of Transportation and Communication, Taiwan based on travel populations and their destinations between 2002 and 2013 [31]. The malaria importation rate was expressed as the number of imported cases per 1,000,000 individuals comprising the travel population. 

The potential for malaria to spread from person to person in a population, a concept that corresponds to the definition of receptivity, is called the basic reproductive number (denoted by R_0_) [10,11]. Most places where malaria has been eliminated have at least some degree of outbreak control in the form of medical attention and outbreak investigation. As a result, the appropriate measure of receptivity is called the reproductive number under control and is denoted R_c _[36]. Each imported malaria case is expected to generate R_c_ new cases, and each one of those cases would also generate R_c_ cases, *etc.* The expected number of locally acquired cases that can be traced back to each imported case is R_c_ in the first generation, R_c_^2^ in the second, and R_c_^n^ in the nth generation. The ratio of locally acquired to imported cases approximates the current level of R_c _[10]. Halting endemic transmission and draining the reservoir requires that R_c _be reduced to less than 1 to prevent malaria from becoming endemic again [37]. All statistical analyses were performed using Stata Statistical Software, Release 10.0 (Stata Corporation, College Station, TX, USA). The accepted level of significance for all analyses was *p* < 0.05. 

## 3. Results

From 2002 to 2013, a total of 229 persons were reported with malaria in Taiwan. Table 1 shows the socio-demographic characteristics of the patients. The mean age was 39.9 years (SD = 13.3), and the median age was 40 years (range: 3 to 70 years). Most of the patients (95%) were older than 18 years of age. The male-to-female ratio was 5.0 to 1. Approximately 62% of the patients did not receive pre-travel medical advice. The reasons for travel were business (68%), visiting friends or relatives (VFR) (17%), tourism (13%), and other (2%). The delay in diagnosis (delay from development of symptoms to the diagnosis of malaria) was less than 7 days for 44%, 8–13 days for 41%, and longer than 14 days for 16%. The highest annual number of malaria cases occurred in 2003 (34 cases) and the lowest number occurred in 2009 (10 cases).

**Table 1:** Socio-demographic characteristics of the study subjects (N = 229).

Variables	Number of Cases	%
**Age **		
<18	11	5
>18	218	95
**Sex **		
Male	191	83
Female	38	17
**Reason for travel**		
Business	156	68
VFR	39	17
Travel	30	13
Other	44 4	2
**Pre-travel medical advice**		
Yes	87	38
No	142	62
**Delay in diagnosis**		
<7 days	98	43
8–13 days	94	41
>14 days	27	12
Unknown	10	4

Notes: VFR: visiting friends and relatives; Delay in diagnosis: delay from development of symptoms to the diagnosis of malaria.

**Figure 1:**
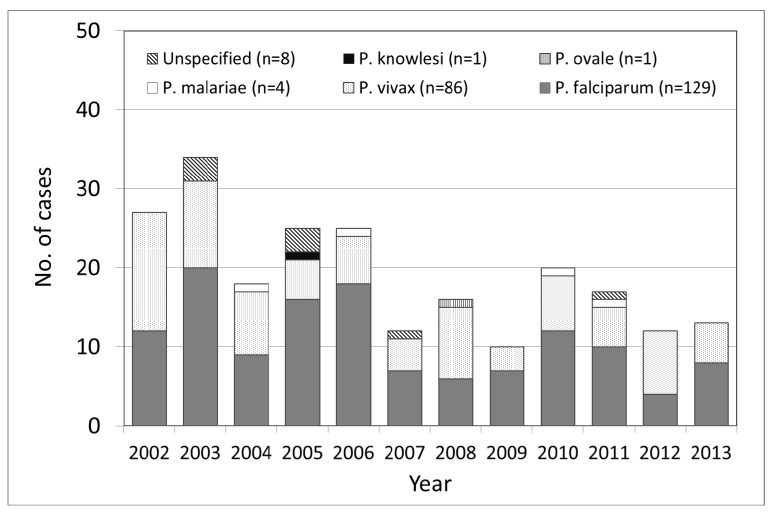
Annual number of imported malaria cases by species in Taiwan.

The number of imported malaria cases varied between 10 and 34 cases per year. Among the 229 cases, the infecting *Plasmodium* species was identified and reported in 221 (97%) cases. *P. falciparum* and *P. vivax* accounted for the majority of infections and were identified in 56% and 38% of the patients, respectively. In addition, one confirmed case of *P. knowlesi* was reported. In eight cases the species remained unidentified (Figure 1). 

Among the 229 cases, 43% were acquired in Africa, and 44% were acquired in Asia. Among the 229 cases for which both the region of acquisition and the infecting species were known, *P. falciparum* accounted for 71% (92/129) of the infections acquired in Sub-Saharan Africa, 22% (28/129) of the infections acquired in Asia, and 7% (9/129) of the infections acquired in Oceania. The infections attributed to *P. vivax* accounted for 3% (3/86) of those acquired in Africa, 78% (67/86) of those acquired in Asia, and 16% (14/86) of those acquired in Oceania (Table 2). 

**Table 2:** The species of malaria reported by region of likely acquisition, 2002–2013.

Region	*P. falciparum*	*P. vivax*	*P. malariae*	*P. ovale*	*P. knowlesi*	Unspecified	Total
**Asia**	**28**	**67**	**1**	**0**	**1**	**4**	**101**
Southeast	23	50			1	4	78
South	2	14					16
Other	3	3	1				7
**Africa**	**92**	**3**	**1**	**1**		**2**	**99**
Central	9			1			10
South	4						4
East	26	3				1	30
West	53		1			1	55
**South America**		**2**					**2**
**Oceania**	**9**	**14**	**2**			**2**	**27**
**Total**	**129**	**86**	**4**	**1**	**1**	**8**	**229**

**Figure 2:**
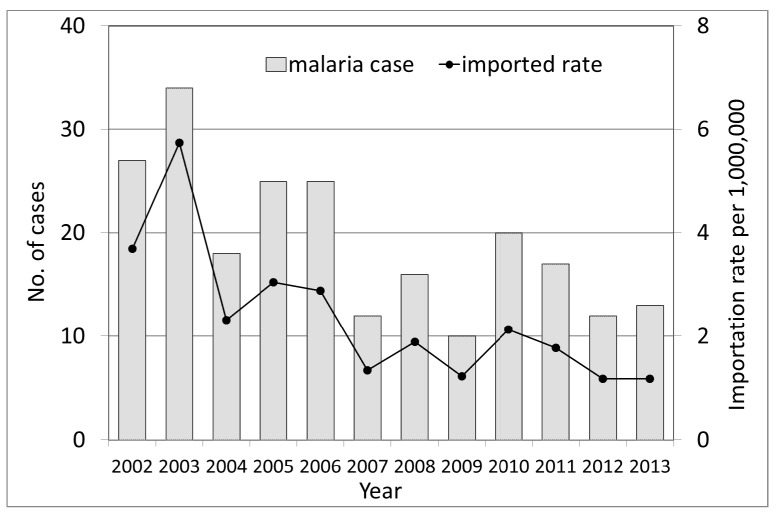
Malaria importation rate and number of imported cases in Taiwan by year.

The use of statistics from the National Tourism Bureau, Ministry of Transportation and Communication enabled a more precise estimation of the number of travelers entering countries where they might be exposed to malaria, and these numbers were used as the denominator for malaria cases acquired in these countries. The annual malaria importation rate changed significantly during the study period (χ^2^ for linear trend = 37.7; *p* < 0.0001), with a reduction in the rate of 37%. The mean annual malaria importation rate was 2.36 per 1,000,000 (range 1.20–5.74) (Figure 2). This result met the minimal requirement of maintaining interruption of malaria transmission, an infection importation rate (IIR) of less than 0.2 per 1,000 population [38]. 

**Figure 3:**
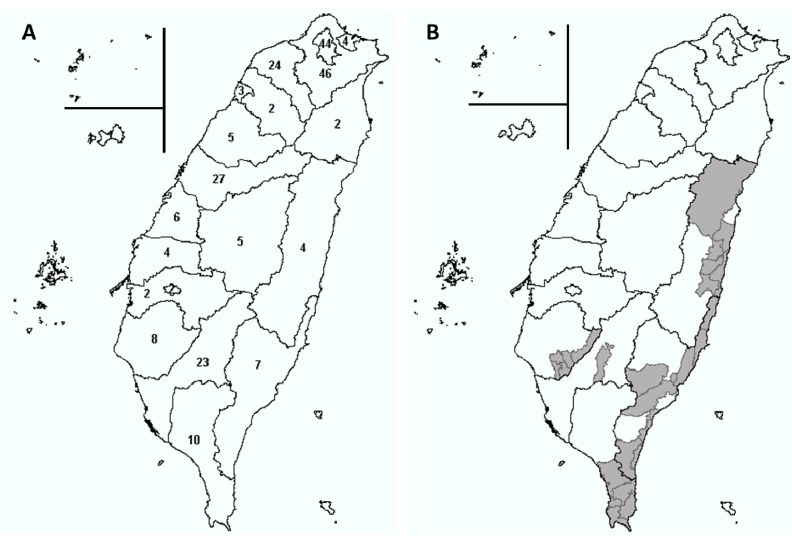
(**A**) A map of imported malaria cases in Taiwan, 2002–2013, and (**B**) distribution of *Anopheles* *minimus* in Taiwan. The grey areas indicate the collection of *An. minimus* adults (at least once) based on the light trap data, 2003–2006.

From 2002 to 2013, all of the 22 counties in Taiwan reported imported malaria cases. Six of the counties accounted for 76% of the reported cases. No secondary transmission was found. A large number of *An. minimus* adults were collected in these counties using light traps during the same or prior year. Of the six counties, four (18%) counties harboring *An. minimus* reported imported cases. All four counties had previously had at least one calendar year in which no imported cases were reported. The remaining 16 counties reported 1–8 imported cases over the 12-year period. No imported cases were reported in any county for <4 weeks during the study period. In general, imported cases tended to cluster in counties in which no *An. minimus* was reported (Figure 3).

We calculated the reproductive number under control (R_c_) to quantify the malaria transmission in Taiwan. During the study period, all of the cases were imported. No secondary cases were found. The ratio of locally acquired cases to imported cases was 0:229. This result met the minimal requirement for malaria elimination [39]. 

## 4. Discussion and Conclusions

During 2002–2013, 229 cases of imported malaria were reported in Taiwan. Out of the cases with a known place of disease acquisition, 44% were acquired in Asia and 43% were acquired in Africa. *P. falciparum* (56%) was the dominant imported species; no fatalities were reported. Imported cases were associated with travel to high-risk malaria-endemic areas, such as Africa, primarily for business or to VFR. More than 60% did not receive pre-travel medical advice. 

In Taiwan, malaria cases are usually imported from Africa and Asia. In general, our findings support those reported in the literature and show that Africa plays a key role in the importation of malaria to industrial countries where malaria is not endemic [40]. In our study, >40% of all imported malaria cases were acquired in Africa.

Although no indigenous case was found in Taiwan in recent decades, Taiwan seems to be vulnerable to the re-establishment of endemic transmission of malaria. The reasons for Taiwan’s vulnerability include the following: Taiwan’s climate, the proximity of human populations to mosquito-laden areas, and the increased number of travelers and immigrants from malaria-endemic countries. Greece has been malaria free since 1974; however, sporadic cases of autochthonous malaria are occasionally reported [41]. The Taiwanese should be aware of their vulnerability to the re-establishment of endemic malaria. However, this study found that the receptivity, vulnerability, and malariogenic potential were below the threshold for the endemic transmission of malaria. In addition, as long as current healthcare, mosquito control, and public health infrastructures remain intact, the re-establishment of endemic areas in Taiwan for malaria remains unlikely. 

In the United Kingdom [42], 191 (0.5%) deaths occurred in 39,302 cases of confirmed malaria between 1987 and 2006. The risk factors for mortality from imported malaria are elderly, tourist, and those presenting in areas in which malaria is seldom seen. In Taiwan, a total of 13 cases of imported malaria have been reported to the Taiwan CDC since 2013, a 52% decrease from the 27 cases reported in 2002 and zero mortality during the study period. Several possible explanations might account for this decrease and zero mortality, including changes in worldwide travel patterns, decreased transmission, changes in case monitoring, and improved prevention interventions and treatment. The decrease is unlikely to be a result of a decrease in travel between Taiwan and malarious countries, because from 2002 to 2013, cumulative travel to and from Taiwan increased annually by an estimated 4% [31]. Chemoprophylaxis is considered appropriate if they followed the national guidelines valid at the time of travel. The chemoprophylaxis in Taiwan is mefloquine or atovaquone/proguqnil or doxicyclin for region where *P. falciparum* malaria is chloroquine-resistant, and autovaquone/proguanil or doxicycline for region where it is mefloquine-resistant [33]. Effective treatment in Taiwan are initiated on the day of diagnosis. The treatment regimens are artemisinin-based combination therapies, in which artesunate plus mefloquine is for *P. falciparum* infection and artesunate plus mefloquine or chloroquine followed by primaquine is for *P. vivax* infection [33]. 

An additional possible explanation for the decrease in the number of Taiwanese cases includes worldwide efforts to decrease the transmission of malaria in many countries where the disease is endemic. These malaria prevention and treatment programs have resulted in increased coverage of effective interventions in some countries and a decrease in associated malaria morbidity [40]. With continued prevention and treatment efforts resulting in reduced transmission in countries with endemic malaria, the number of cases in Taiwan might continue to decrease, but this is uncertain. 

The risk of a traveler acquiring malaria is considered the highest in sub-Saharan Africa and Papua New Guinea, intermediate on the Indian subcontinent, and the lowest in southeast Asia and Latin America [43,44,45]. The numbers assigned to the relative risk in these regions, however, are quite variable [46,47,48]. The total number of travelers is often unknown, so most reports are based on national reporting data and therefore lack a denominator. Thus, assessing risk based on such figures is difficult. The country-specific risk for acquiring malaria varied from 714 per 100,000 travelers in Ghana to 2.5 per 100,000 travelers in Thailand [49]. These data enabled us to conduct a risk analysis for people traveling to malaria-endemic areas. The denominator was based on data collated by the Taiwan Immigration and Tourism infrastructure and thus provided the most representative denominator for travelers to foreign countries. Currently, a number of other denominator sources are used to provide the number of travelers, and a limited comparison was attempted. The arrivals data from the United Nations World Tourism Organization (WTO), one of the most widely used sources for the number of travelers to a country or region, are based on ticket sales [50].

In 2006, Taiwan CDC invited 11 hospitals to establish travel medicine clinics, which provide integrated service over travel-related medicine, health consultation, international travel vaccination and provision of malaria chemoprophylaxis for people having an international travel [51]. However, this study found that more than 60% of travelers to malaria endemic countries in Taiwan did not receive pre-travel advice. According to a previous study [52], the majority (70%) of immigrants returning to their malaria endemic countries of origin did not receive travel information through a pre-travel consultation in Taiwan; more than 40% reported that they did not use measures to prevent insect bites. These behaviors have been suggested to be due to a lack of knowledge of malaria transmission and prevention [53], a belief that malaria is a minor illness, an erroneous trust in lifelong immunity, and the relatively high cost of prophylaxis [3,52,54]. All these data indicate that more educational approaches should be targeted toward travelers who visit and immigrants from malaria endemic areas. 

The Taiwan CDC relies on reporting from each county to compile national data on malaria cases; therefore, clinicians and health-care facilities should report all malaria cases promptly so that annual trends can be assessed and monitored. Taiwan remains at risk for the reintroduction of malaria because of the presence of the mosquito vector and conducive environmental conditions. Therefore, clinicians and health-care facilities should continue to report all malaria cases to their respective county public health authorities. 

*An. minimus* is the primary vector of malaria in Taiwan [15]. Clinicians should be aware that non-falciparum species can cause severe illness and therefore should emphasize the prevention of all types of malaria when counseling patients prior to travel [43]. The differential diagnosis of fever in a person who has returned from travel should always include malaria as one of the primary possibilities. Signs and symptoms of malaria are often nonspecific but typically include fever. Other symptoms include headache, chills, increased sweating, back pain, myalgia, diarrhea, nausea, vomiting, and cough. Prompt diagnosis requires that malaria be included in the dif­ferential diagnosis of illness in a febrile person with a history of travel to a malarious area. Any delay in the diagnosis and treatment of malaria can result in complications, regardless of the effectiveness of the treatment regimen. Although the number of malaria cases has been declining during the past several years in Taiwan [15], the risk for travelers is still evident and should be a concern for physicians who provide pre-travel advice or evaluate a returning traveler with a fever.

The strength of our study is the mosquito and case data, which was combined with additional data regarding the number of trips to destination countries. The limitations of our study include artifacts in the malaria surveillance data and traveler statistics. Malaria surveillance reports may be imprecise with regard to the actual country of origin for the infection because travelers often visit many countries within the region. If a clinical episode develops during travel, the case will not be included in the national surveillance data for a country, further reducing the accuracy of these reports. Underreporting of cases remains a problem in many countries [23]; therefore, our study may underestimate the true incidence of imported malaria in Taiwan.

In conclusion, from 2002 to 2013, the incidence of malaria was lower than the threshold of an epidemic. Endemic malaria transmission was not sustained in any year, in any county, or in Taiwan as a whole. All of the reported malaria cases were associated with importation, and transmission of the disease from these cases was extremely limited. This limited spread indicates a highly sensitive surveillance system and a low vector density (the result of a highly effective malaria eradication program). The sustained elimination of malaria in Taiwan will require this malaria eradication program to be maintained in combination with timely detection of malaria cases, environmental management, vector-control efforts, and an educational approach focused on travelers and immigrants who visit malaria endemic areas. 
